# Which field walking test should be used to assess functional exercise capacity in lung cancer? an observational study

**DOI:** 10.1186/s12890-015-0075-2

**Published:** 2015-08-12

**Authors:** Catherine L. Granger, Linda Denehy, Selina M. Parry, Joel Martin, Tim Dimitriadis, Maeve Sorohan, Louis Irving

**Affiliations:** 1 Department of Physiotherapy, Royal Melbourne Hospital, Grattan Street, Parkville, VIC Australia; 2 Department of Physiotherapy, The University of Melbourne, 161 Barry Street, Parkville, VIC Australia; 3 Institute for Breathing and Sleep, Heidelberg Road, Heidelberg, VIC Australia; 4 Department of Respiratory and Sleep Medicine, Royal Melbourne Hospital, Grattan Street, Parkville, VIC Australia

**Keywords:** Lung cancer, Exercise capacity, Incremental-shuttle walk test, Six-minute walk distance, Endurance shuttle walk test, Cardio-pulmonary exercise test

## Abstract

**Background:**

There is emerging evidence regarding the efficacy of exercise training to improve exercise capacity for individuals with non-small cell lung cancer (NSCLC). Cardiopulmonary exercise testing (CPET) is the gold standard measure of exercise capacity; however this laboratory test has limitations for use in research and clinical practice. Alternative field walking tests are the six-minute walk test (6MWT), incremental-shuttle walk test (ISWT) and endurance-shuttle walk test (ESWT); however there is limited information about their clinimetric properties in NSCLC. Aims: In NSCLC to determine the 1) criterion validity of the 6MWT, ISWT and ESWT against CPET; 2) construct validity of the 6MWT, ISWT and ESWT against measures of function, strength, respiratory function and health-related quality of life (HRQoL); and 3) clinical applicability of the tests.

**Methods:**

Twenty participants (40 % male, mean ± SD age 66.1 ± 6.5 years) with stage I-IIIb NSCLC completed the 6MWT, ISWT, ESWT and CPET within six months of treatment. Testing order was randomised. Additional measures included Eastern Cooperative Oncology Group Performance-Status (ECOG-PS, function), respiratory function, hand-grip dynamometry and HRQoL. Correlations and regression analyses were used to assess relationships.

**Results:**

The ISWT demonstrated criterion validity with a moderate relationship between ISWT distance and CPET peak oxygen consumption (*r* = 0.61, *p* = 0.007). Relationships between CPET and six minute walk distance (6MWD) (*r* = 0.24, *p* = 0.329) or ESWT time (*r* = 0.02, *p* = 0.942) were poor. Moderate construct validity existed for the 6MWD and respiratory function (forced vital capacity % predicted *r* = 0.53, *p* = 0.019; forced expiratory volume in the first second % predicted *r* = 0.55, *p* = 0.015). There were no relationships between the walking tests and measures of function, strength or HRQoL. The ESWT had a ceiling effect with 18 % reaching maximum time. No floor effects were seen in the tests. The mean ± SD time required to perform the individual 6MWT, ISWT and ESWT was 12.8 ± 2.5, 14.7 ± 3.7 and 16.3 ± 5.0 min respectively; in comparison to CPET which was 51.2 ± 12.7 min. Only one assessor was required to perform all field walking tests and no adverse events occurred.

**Conclusions:**

The ISWT is a promising measure of functional exercise capacity in lung cancer. Findings need to be confirmed in a larger sample prior to translation into practice.

## Background

Non-small cell lung cancer (NSCLC) is associated with high disease burden, physical hardship and significant morbidity [1–3]. Functional decline is rapid after diagnosis [4, 5] and impairment in exercise capacity, physical function and health-related quality of life (HRQoL) can ensue [6, 7]. Exercise is a non-pharmacological intervention with great potential benefit for people with NSCLC. Studies to date show that exercise interventions are associated with improvements in exercise capacity for patients with NSCLC [8–10]; however important questions remain as to the most suitable test to assess exercise capacity in both research and clinical settings.

Exercise capacity is “the maximal capacity of an individual to perform aerobic work or maximal oxygen consumption” [11]. The gold standard measure of exercise capacity is cardiopulmonary exercise testing (CPET) used to determine peak oxygen consumption (VO_2_peak) [12]. Symptom-limited CPET is safe in patients with NSCLC [12, 13] and pre-operative VO_2_peak, measured by CPET, is a strong and independent predictor of survival [14]. However CPET has limitations for use in clinical practice and research studies: this laboratory test is expensive, time consuming and requires experienced technicians. In the NSCLC exercise studies published to date, CPET has been used less frequently than alternative field walking tests [8–10].

The six-minute walk test (6MWT), incremental-shuttle walk test (ISWT) and endurance-shuttle walk test (ESWT) are commonly used field walking tests which measure functional exercise capacity [15]. These field walking tests are alternatives to laboratory-based CPET, however they are often criticised and considered not valid or sensitive enough to detect changes in exercise capacity because they may not sufficiently stress the cardiovascular system in individuals without concomitant comorbid disease [16]. Therefore understanding the validity of these tests in NSCLC is vital. Whilst a test may have excellent validity as a measurement tool in one patient group, these findings cannot always be extrapolated to other patient groups [17]. In NSCLC specific features including fatigue, cancer cachexia and loss of peripheral muscle strength can uniquely influence performance [7, 18]. Therefore consideration of the ability of the test to measure what it is intended to measure, that is, how well the data relate to data obtained from the gold standard test (CPET) (*criterion validity*); and how well the test obtains data, as hypothesised, when compared to a test measuring a similar construct (*construct validity*) is important [17, 19]. The 6MWT is the most commonly used field walking test in NSCLC [20] however there is a lack of evidence of its criterion validity in NSCLC [20]. In chronic obstructive pulmonary disease (COPD) there is a moderate relationship between 6MWT and CPET (*r* = 0.59–0.93) [21, 22]. The ISWT and ESWT are less commonly used in NSCLC but have emerged over the last decade as robust measures of exercise capacity in COPD [15, 23, 24]. These tests have the advantage over the 6MWT in that they are externally paced potentially achieving more valid results.

The primary aims of this study were, therefore: 1) to determine the criterion validity of the 6MWT, ISWT and ESWT against CPET in lung cancer. The primary hypothesis was that the 6MWT, ISWT and ESWT would be moderately to strongly correlated (r > 0.5) with VO_2_peak measured by CPET. Secondary aims were to: 2) determine the construct validity of the 6MWT, ISWT and ESWT against measures of physical function, muscle strength, respiratory function and HRQoL; and 3) determine the clinical applicability of the tests. The Strengthening the Reporting of Observational Studies in Epidemiology (STROBE) guidelines [25] and the COnsensus-based Standards for the selection of health status Measurement INstruments (COSMIN) guidelines [19, 26–28] were followed to report this study.

## Methods

### Study design, setting and participants

This was a single centre prospective observational cohort study conducted at a tertiary metropolitan hospital in Melbourne, Australia from July 2013 to December 2014. The study had ethical approval from Melbourne Health Human Research Ethics Committee and written consent was obtained from participants. Participants had histologically confirmed NSCLC; were scheduled to receive or had received treatment within the past six months; and an Eastern Cooperate Oncology Group Performance Status (ECOG-PS) of 0–2. Exclusion criteria included inability to provide consent; cognitive disorder; extensive skeletal or visceral metastases; stage IV disease post-treatment; co-morbidity preventing exercise testing (for example stroke or amputation); insufficient English language skills to complete a questionnaire; or a contra-indication to CPET as recommended by the American Thoracic Society (ATS) [29].

### Procedure

Consecutive patients admitted through the thoracic surgery unit between July 2013 and September 2014 were screened for eligibility. In addition a convenience sample of new patients admitted through the lung oncology outpatient clinics were screened. All patients meeting eligibility criteria were approached.

Assessments were performed at one time-point within two to six months of the participant receiving their last treatment (surgery or chemotherapy) or pre-treatment (if the patient was pre-operative). Each participant completed the 6MWT, ISWT, ESWT and CPET, along with a range of additional tests. Participants completed these assessments over two testing sessions which were no longer than seven days apart. Participants were stable and unchanged in the time between completing the tests and did not receive medical or exercise intervention between testing sessions (participants were specifically asked this between testing sessions). In between tests participants were given a rest to ensure they had recovered (assessed by return to resting heart rate) prior to completing the next test. The rest was a minimum of 15 min.

The order of testing was randomised to account for any order effect. Due to the fact that the ESWT cannot be completed without a prior ISWT (as the ESWT speed is set by the results of the ISWT), the ISWT and ESWT were considered as a block for randomisation. Simple randomisation was undertaken using an off-site computer generated random number table and sequentially numbered sealed opaque envelopes prepared by personnel not involved in the study. For each consented participant the next consecutive assignment envelope was opened and the study coordinator was notified of the allocation of testing order. Assessors were not blinded to order allocation, but participants were not made aware of the testing order prior to completing tests.

### Field walking tests

#### Six minute walk test

The 6MWT is a self-paced field walking test which measures the distance in meters (m) an individual can walk along a flat straight corridor 30 m long in six minutes [15]. The 6MWT was conducted according to published ATS and European Respiratory Society (ERS) recommendations [15, 30]. Duplicate tests were completed to account for the learning effect and the best test result was used in analyses [15]. In patients with COPD the 6WMT is reliable (intra-class correlation coefficients [ICCs] = 0.82-0.99), valid (CPET VO_2_peak *r* = 0.4-0.6) and sensitive to change [15, 31].

#### Incremental shuttle walk test (Department of Respiratory Medicine, Glenfield Hospital, Leicester, UK)

The ISWT is an externally-paced incremental field walking test [15, 23]. The test measures the distance in m an individual can walk around a 10 m shuttle course paced according to an incremental speed dictated by an audio recording [23]. The test speed increases every minute and the test finishes when the participant can no longer maintain the desired speed. The starting (lowest) speed is 0.50 m/s and final (highest) speed is 2.37 m/s; with a maximum possible distance achieved of 1020 m. Duplicate tests were completed to account for the learning effect and the best test result was used in analyses [15]. The test result was also used to predict VO_2_peak using published equations [23, 24]. In COPD the ISWT is reliable (ICC = 0.88), valid (CPET VO_2_peak *r* = 0.75-0.88) and sensitive to change [15, 31].

#### Endurance Shuttle Walk Test (Department of Respiratory Medicine, Glenfield Hospital, Leicester, UK)

The ESWT is an externally-paced field walking test which measures the time in seconds an individual can walk at a pre-set speed [23]. The ESWT is similar to the ISWT however the walking speed remains constant throughout the test (set at 85 % of maximum walking performance from the ISWT) [23]. The participant walks for as long as possible up to a maximum of 20 min (patients are not made aware of the time limit). A single ESWT was performed as duplicate tests do not appear necessary to account for any learning effect [15, 31].

Throughout the tests oxygen saturation and heart rate were monitored using a portable pulse oximeter. Dyspnoea and leg fatigue were measured pre and post-test on the 0–10 modified BORG scale [32]. The differences between pre-test BORG scores (including duplicate 6MWT/ISWT) were assessed to determine participant stability between testing. The minimal important difference in BORG dyspnoea for patients with COPD is 1 point out of 10 [33]. The time and number of staff required to conduct each test was recorded as well as any adverse events.

### Additional measures

Cardiopulmonary Exercise Testing was performed to determine VO_2_peak. Prior to conducting CPET, respiratory function tests (spirometry) were performed as recommended and these were conducted according to the ATS and ERS guidelines [34, 35]. Measurements were made using Sensormedics Vmax Encore, 22D (Sensormedics, Yorba Linda, Ca, USA). Cardiopulmonary exercise testing was performed using a Sensormedics Vmax Encore, 229D (Sensormedics, Yorba Linda, Ca, USA) exercise system and a Ergoline Via Sprint 150P cycle ergometer (Lindenstrabe 5, 72475 Bitz, Germany). A 12-lead electrocardiography was monitored using a GE Cardiosoft Version 6.51 electrocardiography system (GE Healthcare, GE Marquette Medical Systems, 8200 West Tower Ave, Milwaukee, WI 53223. USA). Two minutes of rest data were collected prior to an incremental test with the workload increasing every minute. The workload increments were selected using the equations from Jones [36] and the test continued until symptomatic limitation. The cycle exercise test data were calculated using 30-s averages. Symptomatic levels of breathlessness in combination with participant data (heart rate) were used to establish if the test was maximal. Percentage of predicted VO_2_peak was calculated according to normative equations by Jones [36]. The time required to conduct the test (including initial spirometry tests, CPET set-up and CPET instructions) was recorded.

Physical function was measured using the ECOG-PS rated by the participant [37]. Participants were also asked if they had any limitations to walking as far as they needed to. Hand-grip strength was measured using a Jamar hydraulic hand-grip dynamometer, and was taken as the best of three measures [38]. Health-related quality of life was measured using the European Organization for the Research and Treatment of Cancer questionnaire and lung cancer module (EORTC QLQ-C30-LC13) [8, 39]. Medical and social demographics were obtained including age, sex, body mass index, cancer stage, histological type, treatment type/status and smoking history. Comorbidities were scored using the simplified Colinet comorbidity score [40].

### Sample size

For 80 % power and a significant difference (alpha two tailed 0.05) between the correlation coefficients of the null hypothesis (rho_0_ = 0.50) and alternative hypothesis (rho_1_ = 0.80) (95 % CI = 0.50-0.93) 16 participants were required. To account for an estimated 25 % drop out rate and missing data the sample size was increased to 20.

### Statistical analyses

Data were analysed through SPSS Windows Version 22.0 (SPSS, Chicago, IL, USA). Data were assessed for normality using the Kolmogorov-Smirnov statistic. Parametric data are presented as mean and standard deviation (SD), and non-parametric data are presented as median and inter-quartile range (IQR). Participant stability between testing was assessed using paired t-tests to examine differences in baseline (pre-test) dyspnoea and leg fatigue before each test (6MWTx2, ISWTx2, ESWT and CPET).

Pearson’s correlation coefficient was used to assess the bivariate relationships between test scores (6MWT-m, ISWT distance-m, ESWT time-seconds and CPET VO_2_peak-ml/kg/min) [41]. Coefficients were interpreted as: little (0.00–0.25), fair (0.25–0.50), moderate (0.50–0.75) and strong association (0.75–1.0) [17]. Simple linear regression was used to further explore relationships between the field tests and CPET VO_2_peak. The CPET VO_2_peak was the dependent variable and 6MWT, ISWT distance or ESWT time were the independent variables. Potential covariates were medical and social demographics (listed in Table 1), physical function, hand-grip strength, respiratory function and HRQoL. Potential covariates with significant univariate correlation with the outcome of interest (CPET VO_2_peak) were included in the model if collinearity was not identified. Alpha was set at 0.05 for all analyses.

**Table 1:** Medical and social demographics (*n* = 20)

Variable	Mean ± SD or n (%)
Age, years	66.1 ± 6.5
Gender, male	8 (40.0 %)
Body mass index, kg/m^2^	28.5 ± 4.0
Colinet comorbidity score, median [IQR]	9.0 [8.0 – 10.0]
COPD diagnosis	7 (35.0 %)
Smoking status	
Never smoker	2 (10.5 %)
Current smoker	2 (10.5 %)
Ex-smoker	15 (78.9 %)
Unknown	[1]
Smoking pack year history	38.4 ± 19.6
Lung cancer stage	
Stage I	12 (60.0 %)
Stage II	5 (25.0 %)
Stage III	3 (15.0 %)
Lung cancer histological type	
Adenocarcinoma	15 (75.0 %)
Squamous cell carcinoma	2 (10.0 %)
Other	3 (15.0 %)
Treatment status at time of testing	
Pre-treatment	3 (15.0 %)
Post-surgery only	12 (60.0 %)
Post-surgery and chemotherapy	5 (25.0 %)
Type of thoracic surgery	
Lobectomy	14 (70.0 %)
Pneumonectomy	3 (15.0 %)
N/A (pre-operative)	3 (15.0 %)
Time since last treatment (surgery or chemotherapy), days^a^	134.9 ± 43.9
Use of a gait aid	
No	20 (100 %)
Self-reported limitation to walking	4 (20 %)
ECOG-PS, patient rated	
0, fully active	11 (57.9 %)
1, walking but only light work	8 (42.1 %)
Unknown	[1]
Hand-grip strength, kilograms	14.8 ± 7.4
Respiratory function	
FEV_1,_ litres	1.9 ± 0.4
FEV_1_ % predicted	80.6 ± 16.0
FVC, litres	3.0 ± 0.8
FVC, % predicted	101.8 ± 22.7
Physical activity levels	
Sedentary (0 min/week)	8 (40.0 %)
Insufficient (1–149 min/week)	7 (35.0 %)
Sufficient (150+ minutes/week)	5 (25.0 %)
Time spent watching television, hours/day, median [IQR]	3.0 [2.2 – 5.4]
HRQoL	
Global quality of life domain	76.7 ± 17.9
Physical function domain	77.5 ± 14.5
Social situation	
Home with family	10 (50.0 %)
Home alone, independent	8 (40.0 %)
Retirement village	2 (10.0 %)
Employment status	
Working full time	1 (5.0 %)
Working part time	1 (5.0 %)
Temporary or sick leave	1 (5.0 %)
Not employed	3 (15.0 %)
Retired	14 (70.0 %)
Highest level of education obtained	
PhD or Master’s degree	1 (5.0 %)
Bachelor’s degree	2 (10.0 %)
Trade or community certificate	2 (10.0 %)
Completed secondary school	4 (20.0 %)
Some secondary school	11 (55.0 %)

Abbreviations: *%* percent; *COPD* Chronic Obstructive Pulmonary Disease; *ECOG-PS* Eastern Cooperate Oncology Group- Performance status; *IQR* inter-quartile range; *kg* kilograms; *m* meters; *n* number; *PhD* Doctor of Philosophy; *SD* standard deviation

^a^only participants who were post-treatment included

Floor and ceiling effects of the tests were determined using the percentage of occasions when participants scored the lowest (6MWT = 0 m, ISWT = 0 m, ESWT = 0 s) or highest result (ISWT = 1020 m, ESWT = 1200 s) possible for the tests respectively.

## Results

**Fig. 1:**
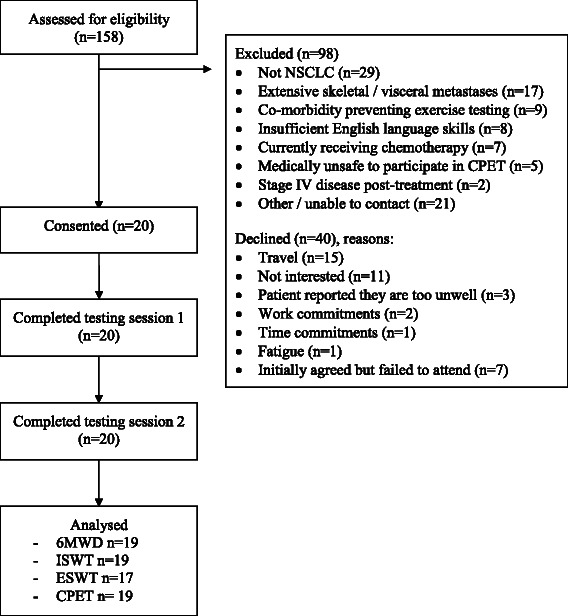
Consort diagram. Abbreviations: *6MWD* six minute walk distance; *CPET* cardio-pulmonary exercise test; *ESWT* endurance shuttle walk test; *ISWT* incremental shuttle walk test; *NSCLC* non-small cell lung cancer

Between August 2013 and December 2014, 158 patients were screened, of whom 38 % (n = 60/158) were eligible and approached (Fig. 1). The consent rate was 33 % (n = 20/60). The main reasons for decline were “too far to travel to the hospital for testing” 37 % (n = 15/40) or “not interested” 27 % (n = 11/40). Twenty patients with lung cancer were studied (Fig. 1).

The characteristics of the cohort studied are reported in Table 1. For the 6MWT, ISWT and CPET data were available for 95 % (n = 19) of the cohort and for the ESWT data were available for 85 % (n = 17). Data were not missing from the same 19 individuals for the 6MWT, ISWT and CPET. A technical failure of equipment during one CPET resulted in missing data from one of the 20 participants, although all 20 participants undertook CPET testing. The median [IQR] time between the testing sessions was 2.5 [0–5.7] days. In this time participants were stable and unchanged: there was no statistically significant difference in baseline (pre-test) dyspnoea between each test (*p* > 0.05). There was a small statistically significant difference between baseline leg fatigue for the first and second 6MWT (mean difference [95 % CI] = 0.5 [0.1-1.0]points, *p* = 0.019) although this difference is not likely to be clinically significant [33]; and between the first 6MWT and the ESWT (mean difference [95 % CI] =1.7 [0.4-3.0]points, *p* = 0.015). There were no other differences in baseline leg fatigue between tests (*p* > 0.05).

### Criterion validity

**Fig. 2:**
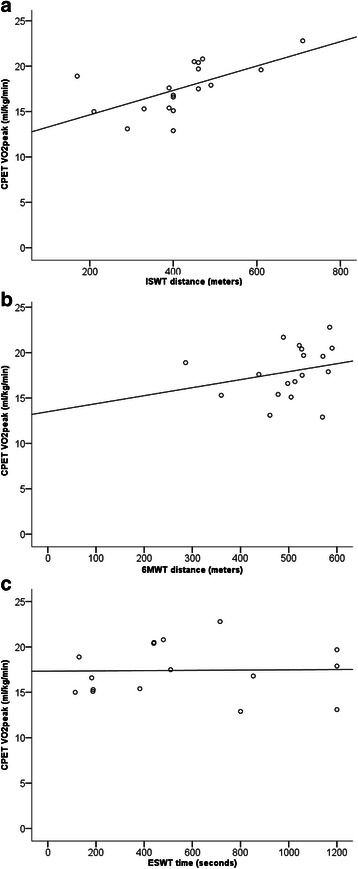
Relationship between ISWT (**a**), 6MWD (**b**) and ESWT (**c**) with CPET VO_2_peak. Abbreviations: *6MWT* six minute walk test; *CPET* cardio-pulmonary exercise test; *ESWT* endurance shuttle walk test; *ISWT* incremental shuttle walk test; *kg* kilograms; *min* minutes; *ml* millilitres; *VO*_*2*_*peak* peak oxygen consumption

The ISWT exhibited moderated criterion validity with CPET VO_2_peak (*r* = 0.61 [95 % CI = 0.20-0.88] *p* = 0.007) (Fig. 2). There was a moderate relationship between ISWT predicted VO_2_peak and CPET VO_2_peak (ml/kg/min) (*r* = 0.61 [95 % CI = 0.16-0.88] *p* = 0.008); however the ISWT tended to under-predict VO_2_peak in comparison to VO_2_peak measured using CPET (Table 2). Simple linear regression demonstrated the ISWT and forced expiratory volume in the first second (FEV_1_) to be significant determinants of CPET VO_2_peak together explaining 59 % of the variance (ISWT B coeff = 0.55, FEV_1_ B coeff = 0.47, *p* = 0.001). The 6MWT and ESWT exhibited a poor relationship with CPET VO_2_peak: 6MWD (*r* = 0.24 [95 % CI = −0.12-0.69] *p* = 0.329) and ESWT (*r* = 0.02 [95 % CI = −0.43-0.53] *p* = 0.942) (Fig. 2).

**Table 2:** Test results

Variable	Mean ± SD
Field walking tests	
6MWD, m	499.0 ± 78
ISWT distance, m	410.0 ± 124.9
ISWT predicted VO_2_peak, ml/kg/min	14.4 ± 3.1
ESWT time, seconds	581.4 ± 383.5
ESWT level, completed	11 ± 3
CPET	
Resting data	
Heart rate, beats/min	90.7 ± 15.4
Systolic blood pressure, mmHg	127.6 ± 15.2
Diastolic blood pressure, mmHg	78.7 ± 12.4
Oxygen saturation, %	97.2 ± 1.3
Peak data	
Heart rate, beats/min	131.2 ± 19.1
Systolic blood pressure, mmHg	167.9 ± 22.9
Diastolic blood pressure, mmHg	92.5 ± 19.9
Oxygen saturation, %	94.2 ± 6.3
VO_2_peak, ml/kg/min	17.8 ± 2.8
VO_2_peak, ml/kg/min, % predicted	76.0 ± 14.3
VO_2_peak, L/min	1.4 ± 0.4
VO_2_peak, L/min, % predicted	74.9 ± 18.4
VE, L/min	59.6 ± 15.5
Work	86.2 ± 33.9

Abbreviations: *%* percent; *6MWD* six minute walk distance; *CPET* cardio-pulmonary exercise test; *ESWT* endurance shuttle walk test; *ISWT* incremental shuttle walk test; *kg* kilograms; *L* litres; *min* minutes; *ml* millilitres; *mmHg* millimetre of mercury; *SD* standard deviation; *VO*_*2*_*peak* peak oxygen consumption

### Construct validity

There was a strong relationship between the 6MWT and ISWT (*r* = 0.80 [95 % CI = 0.64-0.93] *p* < 0.005). Weaker relationships existed between the ESWT and the 6MWT (*r* = 0.42 [95 % CI = −0.11-0.73] *p* = 0.101) or ISWT (*r* = 0.31 [95 % CI = −0.22-0.65] *p* = 0.224).

The 6MWT exhibited moderate construct validity with respiratory function (forced vital capacity % predicted *r* = 0.53 [95 % CI = 0.24-0.86] *p* = 0.019; FEV_1_ % predicted *r* = 0.55 [95 % CI = 0.30-0.76] *p* = 0.015). Small non-significant relationships were seen between FEV_1_ % predicted and both the ISWT (*r* = 0.43 [95 % CI = 0.26-0.68] *p* = 0.065) and ESWT (*r* = 0.45 [95 % CI = −0.11-0.80] *p* = 0.067).

Lower pain levels were associated with better performance in the ESWT (*r* = 0.72 [95 % CI = 0.37-0.96] *p* = 0.002). There were no relationships between ECOG-PS or the EORTC-QLQ-C30 physical function domain score and performance in the field walking tests or CPET. There were no relationships between hand-grip strength or HRQoL and the field walking tests.

All three field walking tests could discriminate between people who reported having walking limitations (*p* < 0.05), which was not the case for CPET. The mean difference [95 % CI] between people with self-rated walking limitations on the 6MWT, ISWT and ESWT were 163.5 [97.9-229.2]m, 171.0 [46.0-296.0]m and 531.1 [80.6-981.7]seconds respectively.

### Clinical applicability

Tests results are provided in Table 2. The six minute walk distance (6MWD) ranged from 286-590 m. No floor or ceiling effect was observed. From pre to post-test there were statistically significant increases in dyspnoea (mean difference [95 % CI] = 2.5 [1.9-3.2] points, *p* < 0.0005) and leg fatigue (mean difference [95 % CI] = 1.7 [0.6-2.7]points, *p* = 0.003) (Table 3). In comparison the increase in dyspnoea and leg fatigue from pre to post CPET were mean difference [95 % CI] 4.2 [3.0-5.3]points (*p* < 0.005) and 4.9 [3.6-6.3]points (*p* < 0.005) respectively (Table 3).

**Table 3:** Change in BORG scores with each test

Test	Pre-test mean ± SD	Post-test mean ± SD	Mean difference (95 % CI), *p* value
Dyspnoea			
6MWT	0.9 ± 1.1	3.4 ± 1.7	2.5 (1.9-3.2), *p* < 0.0005
ISWT	0.6 ± 0.7	4.2 ± 2.1	3.7 (2.7-4.6), *p* < 0.0005
ESWT	0.4 ± 0.6	4.3 ± 2.7	3.8 (2.5-5.2), *p* < 0.0005
CPET	0.7 ± 1.0	4.9 ± 1.9	4.2 (3.0-5.3), *p* < 0.0005
Leg fatigue			
6MWT	1.1 ± 1.7	2.7 ± 2.6	1.7 (0.6-2.7), *p* = 0.003
ISWT	1.0 ± 1.4	3.5 ± 2.8	2.6 (1.5-3.6), *p* < 0.0005
ESWT	1.0 ± 1.3	3.7 ± 2.6	2.8 (1.5-4.1), *p* < 0.0005
CPET	0.7 ± 0.9	5.6 ± 3.1	4.9 (3.6-6.3), *p* < 0.0005

Abbreviations: *6MWT* six minute walk test; *CPET* cardio-pulmonary exercise test; *CI* confidence intervals; *ESWT* endurance shuttle walk test; *ISWT* incremental shuttle walk test; *SD* standard deviation

The ISWT distance ranged from 170-710 m, and the predicted VO_2_peak from this test ranged from 8.4-21.9 ml/kg/min. No floor or ceiling effects were observed. From pre to post-test there were statistically significant increases in dyspnoea (mean difference [95 % CI] = 3.7 [2.7-4.6]points, *p* < 0.0005) and leg fatigue (mean difference [95 % CI] = 2.6 [1.5-3.6]points, *p* < 0.0005) (Table 3).

The ESWT time ranged from 114-1200s. The ESWT was performed on levels ranging from 6–16. A ceiling effect was seen with 17.6 % (n = 3/17) of participants reaching the end of the test (1200 s). No floor effect was seen. From pre to post-test there were statistically significant increases in dyspnoea (mean difference [95 % CI] = 3.8 [2.5-5.2]points, *p* < 0.0005) and leg fatigue (mean difference [95 % CI] = 2.8 [1.5-4.1] *p* < 0.0005 (Table 3).

The mean ± SD time required to perform the individual 6MWT, ISWT and ESWT were 12.8 ± 2.5 min, 14.7 ± 3.7 min and 16.3 ± 5.0 min respectively. In comparison the time required to perform the CPET was 51.2 ± 12.7 min. Only one assessor was required to perform the field walking tests, whereas the CPET required two to three assessors. No adverse events occurred.

## Discussion

This is the first study to compare the validity of three common field walking tests against the gold standard measure of exercise capacity, CPET, in NSCLC. Our results demonstrate that in a cohort with operable stage I-IIIb NSCLC the ISWT demonstrated greater criterion validity than the 6MWT and ESWT with CPET. This is likely due to the fact that the ISWT is incremental in a similar manner to CPET, with the potential to test patients closer to their maximal exercise capacity. In contrast the 6MWD is performed at the participants own pace and the ESWT is performed at a constant sub-maximal speed. Our results are consistent with the only other study examining the criterion validity of the ISWT in lung cancer which also found the ISWT to be related to CPET (*r* = 0.67) [42]. Studies have demonstrated that pre-operative ISWT is a predictor of post-operative survival in NSCLC [42]; our study adds further to the literature in supporting the use of the ISWT in lung cancer.

Our study has shown that the ISWT and 6MWT have excellent convergent validity suggesting they measure a similar construct to each other; however only the ISWT is moderately related to CPET (and 6MWT is poorly related). The CPET measures a different construct to the field walking tests and this may explain the discrepancy between CPET and the field walking tests. The fact that the ISWT is incremental may explain its closer association to CPET. The ISWT and 6MWT are functional tests, which measure functional exercise capacity and walking capacity [31]. Results demonstrate that the field walking tests are able to discriminate between participants with self-reported walking limitations which CPET cannot. Similarly in COPD performance in the 6MWT is closely related to activities of daily living [43]. On the other hand CPET is a measure of cardiorespiratory fitness. The main outcome of interest from CPET is VO_2_peak, which is calculated as the product of cardiac output and arteriovenous oxygen difference [16]. It is commonly performed in lung cancer to determine physiological status and risk stratification of patients being considered for lung resection [44]. Our findings suggest that CPET and the field walking tests are not interchangeable. There are times when CPET may be preferred over the ISWT or 6WMD as an outcome measure; for example in the case of prehabilitation when patients may undergo aerobic exercise training with the primary intent to improve cardiorespiratory fitness to be fit enough for lung resection. Alternatively, exercise rehabilitation programs for patients with advanced lung cancer may use a field walking test as the measure of interest when the primary intent is to improve functional exercise capacity.

There are many advantages to non-laboratory field walking tests: in a clinical setting these tests can be performed quickly, simply (with little expertise required) and relatively cheaply (little equipment required). Our results show that all three field walking tests are quick (less than 16 min), safe (no adverse events) and require only one assessor. This means that these tests can easily be performed by clinicians in their own setting, such as an oncology ward, inpatient rehabilitation class or community rehabilitation setting to assess, re-assess or monitor their patients. In a research study the ability to perform measurement with few costs, minimal training of research assistants and little time burden for participants (and potential drop-outs) is extremely advantageous. Although the reproducibility of these field walking tests (6MWT, ISWT, ESWT) has not been established specifically in the lung cancer population, these tests have been demonstrated to have high reproducibility in individuals with chronic respiratory disease [31, 45]. It has been shown in non-lung cancer respiratory populations that there is a learning effect for the 6MWT and ISWT, and therefore two tests are recommended according to the ATS guidelines [15, 31, 45]. This methodology was followed within this study.

The 6MWT is currently the most commonly used field walking test in NSCLC [20]. It is likely that its favourable use stems from the COPD literature. The 6MWT is an extremely robust measure of functional exercise capacity in COPD and has strong, well established clinimetric properties, including excellent reliability (ICC = 0.82-0.99), moderate validity (CPET VO_2_peak *r* = 0.4-0.6) and sensitivity to change [15, 31]. In COPD the 6MWT is the standard measure to evaluate pulmonary rehabilitation efficacy [15]. Despite the fact that many patients with lung cancer have COPD as a comorbidity (35 % in our cohort), cancer cachexia and symptom clusters such as fatigue, dyspnoea and pain [46, 47] are unique in lung cancer; consequently patients with lung cancer may perform differently to those with COPD. In NSCLC there is little published evidence regarding the clinimetric properties of the 6MWT. Previous research has demonstrated the good predictive validity of the 6MWT for post-operative outcomes in NSCLC [48, 49] and survival in patients with advanced NSCLC [50, 51]. The BODE index (which includes the 6MWT) is a strong independent predictor of survival in inoperable NSCLC [52]. Given the frequent use of the 6MWD in lung cancer, further research is required to confirm our findings and help to establish if use of the ISWT could be substituted for the 6MWT in certain subgroups of NSCLC. In this study we found the ISWT under-predicted VO_2_peak in comparison to the CPET, albeit it is within a small study sample. We hypothesise that this may be due to the formula for prediction of VO_2_peak not specifically being derived from a lung cancer population for the ISWT and potentially that the ISWT may not be a maximal test of functional capacity in all patients.

Our study did not find the 6MWT or ISWT to be closely related to the ESWT. The ESWT was originally developed to examine endurance capacity rather than functional exercise capacity [23]. We are unaware of other studies which have examined the clinimetric properties of the ESWT in lung cancer; based on our results we do not recommend this test at present to examine exercise capacity.

Muscle strength is a strong predictor of exercise performance in COPD [53, 54] and peripheral muscle power is related to performance in the ISWT in thoracic cancer (*r* = 0.39) [18]. Previous research has established that hand-grip strength is related to physical function (*r* = 0.44) and performance status (*r* = 0.50) in patients with heterogeneous cancers [55]. We hypothesized that hand-grip strength, as a surrogate measure for whole body strength, may be related to exercise capacity in NSCLC. Our finding that hand-grip strength was poorly related to performance in the field walking tests may be explained by the fact we measured hand-grip strength instead of quadriceps muscle strength: future research is required to explore this further.

Our study is limited by a small sample size and single centre design. Participants had operable disease and were a relatively fit cohort (ECOG-PS ≤ 2 rated by the physician, only two individuals had VO_2_peak < 15 ml/kg/min), therefore findings cannot be extrapolated to patients with inoperable disease or those with poorer functional status or exercise capacity. Despite randomisation of testing order and rest periods between tests, there was a small statistically significant difference in baseline leg fatigue between the first 6MWT and ESWT; this may have led to poorer performance in the ESWT.

## Conclusions

The ISWT is a valid and clinically applicable measure of functional exercise capacity in operable NSCLC. The ISWT has moderate criterion validity with CPET VO_2_peak; is quick and safe to perform; and does not appear to have a floor or ceiling effect in this population. Both the 6MWT and ESWT have poor validity with CPET VO_2_peak. The ESWT has a ceiling effect and is not currently recommend for use in NSCLC. The ISWT appears to be the most promising field measure of exercise capacity however findings need to be confirmed in larger sample prior to translation into practice.

## Abbreviations

6MWD: six minute walk distance
6MWT: six minute walk test
%: percent
ATS: American Thoracic Society
CPET: cardio-pulmonary exercise test
CI: confidence intervals
COPD: chronic obstructive pulmonary disease
COSMIN: Consensus-based standards for the selection of health status measurement instruments
ECOG-PS: Eastern cooperate oncology group- performance status
ERS: European Respiratory Society
ESWT: endurance shuttle walk test
HRQoL: health-related quality of life
IQR: inter-quartile range
ISWT: incremental shuttle walk test
kg: kilograms
L: litres
m: meters
mHg: millimetre of mercury
min: minutes
ml: millilitre
NSCLC: non-small cell lung cancer
PhD: doctor of philosophy
SD: standard deviation
STROBE: Strengthening the reporting of observational studies in epidemiology
VO_2_peak: peak oxygen consumption
